# Rifampicin for Continuation Phase Tuberculosis Treatment in Uganda: A Cost-Effectiveness Analysis

**DOI:** 10.1371/journal.pone.0039187

**Published:** 2012-06-18

**Authors:** Yukari C. Manabe, Sabine M. Hermans, Mohammed Lamorde, Barbara Castelnuovo, C. Daniel Mullins, Andreas Kuznik

**Affiliations:** 1 Infectious Diseases Institute, Makerere University College of Health Sciences, Kampala, Uganda; 2 Division of Infectious Diseases, Department of Medicine, Johns Hopkins School of Medicine, Baltimore, Maryland, United States of America; 3 Department of Global Health, Academic Medical Center, University of Amsterdam, Amsterdam Institute for Global Health and Development, Amsterdam, The Netherlands; 4 Department of Pharmacology and Therapeutics, Trinity College Dublin, Dublin, Ireland; 5 University of Maryland, Baltimore, Maryland, United States of America; 6 Pfizer Inc., New York, New York, United States of America; McGill University, Canada

## Abstract

**Background:**

In Uganda, isoniazid plus ethambutol is used for 6 months (6HE) during the continuation treatment phase of new tuberculosis (TB) cases. However, the World Health Organization (WHO) recommends using isoniazid plus rifampicin for 4 months (4HR) instead of 6HE. We compared the impact of a continuation phase using 6HE or 4HR on total cost and expected mortality from the perspective of the Ugandan national health system.

**Methodology/Principal Findings:**

Treatment costs and outcomes were determined by decision analysis. Median daily drug price was US$0.115 for HR and US$0.069 for HE. TB treatment failure or relapse and mortality rates associated with 6HE vs. 4HR were obtained from randomized trials and systematic reviews for HIV-negative (46% of TB cases; failure/relapse –6HE: 10.4% vs. 4HR: 5.2%; mortality –6HE: 5.6% vs. 4HR: 3.5%) and HIV-positive patients (54% of TB cases; failure or relapse –6HE: 13.7% vs. 4HR: 12.4%; mortality –6HE: 16.6% vs. 4HR: 10.5%). When the initial treatment is not successful, retreatment involves an additional 8-month drug-regimen at a cost of $110.70. The model predicted a mortality rate of 13.3% for patients treated with 6HE and 8.8% for 4HR; average treatment cost per patient was predicted at $26.07 for 6HE and $23.64 for 4HR. These results were robust to the inclusion of MDR-TB as an additional outcome after treatment failure or relapse.

**Conclusions/Significance:**

Combination therapy with 4HR in the continuation phase dominates 6HE as it is associated with both lower expected costs and lower expected mortality. These data support the WHO recommendation to transition to a continuation phase comprising 4HR.

## Introduction

The public health burden of tuberculosis (TB) disproportionately affects developing countries. Limited resources in these countries increase the risk of suboptimal treatment and necessitate financial analyses that inform health policy decisions. HIV increases the risk for primary active disease and reactivation TB, and has compounded the TB epidemic in sub-Saharan Africa which bears 80% of the HIV-TB co-infected patient burden [1]. Curative TB treatment requires a prolonged course of a combination of antibiotics that is divided into a 4-drug, 2-month intensive phase of rifampicin, isoniazid, pyrazinamide and ethambutol (2RHZE), followed by a continuation phase of 2 drugs administered for 4–6 months depending on the choice of regimen [2]. Directly observed therapy, short course (DOTS), introduced in 1994, emphasized governmental commitment and ownership of TB control programs, TB case detection, standardized short-course chemotherapy, regular drug supply and implementation of monitoring systems [3]. Initially, six months of isoniazid plus thiacetazone (6HA) was recommended by the World Health Organization (WHO) for the continuation phase rather than a rifampicin-containing regimen. This was to preserve sensitivity to rifampicin for retreatment. Due to severe skin reactions observed in HIV-positive individuals [4], the WHO recommended that national TB programs change the continuation phase to 6 months of isoniazid plus ethambutol (6HE) in 1991 [5]. In 2003, as evidence mounted in support of rifampicin throughout TB treatment, the WHO recommended one of two approaches for the continuation phase of TB treatment; 6 months of isoniazid plus ethambutol (6HE) or 4 months of isoniazid plus rifampicin (4HR) for programs that could assure good monitoring of adherence with DOT [6]. As a result of clinical trial evidence of the superiority of 4HR over 6HE in the continuation phase with significantly lower relapse rates (5% compared to 10%) [7], the WHO recommended that TB programs transition to a continuation phase of 4HR. In 2008, 23 countries including 4 high burden countries (13% of the global TB burden) were still using only 2 months of rifampicin. In 2009, the WHO released preliminary guidelines that reinforced this position and, in 2010, final guidance recommending the phase-out of 6HE in the continuation phase was published [8]. In the 2011 WHO Global TB report, 3 high-burden countries still reported using rifampicin for only 2 months (Nigeria, Afghanistan, Pakistan) [1]. Although Uganda is listed as using rifampicin throughout treatment, this option is made available only to children in its national guidelines [9].

**Figure 1:**
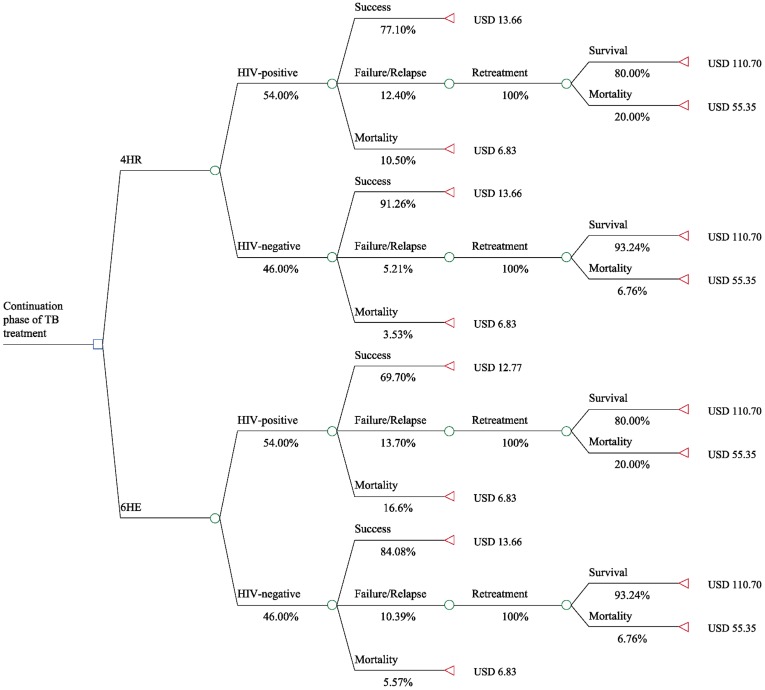
Model #1.

Comparative effectiveness and cost analyses of alternative treatment strategies using 6HE versus 4HR inform health policy decisions for TB programs in resource-poor settings such as Uganda. Therefore, we sought to model the impact of the use of 4HR in the continuation phase of TB treatment compared to the use of 6EH on expected patient mortality and costs with and without inclusion of the risk estimates for multi-drug resistance. To that end, we developed two decision analytic models to evaluate healthcare-related costs in a hypothetical cohort of Ugandan TB patients initiated on either a continuation phase of 6HE or the alternative regimen of 4HR.

**Table 1:** Treatment of new TB cases.

Treatment of new TB cases	6HE	4HR	Difference	Source Data
Efficacy				
*HIV-negative patients*				
Treatment Success	84.0%	91.3%	7.2%	Jindani [7], Nunn [11] a
Treatment Failure/Relapse	10.4%	5.2%	5.2%	Jindani [7], Nunn [11] a
Treatment Mortality	5.6%	3.5%	2.0%	Jindani [7], Nunn [11] a
*HIV-positive patients*				
Treatment Success	69.7%	77.1%	7.4%	Khan [21] a
Treatment Failure/Relapse	13.7%	12.4%	1.3%	Khan [21]
Treatment Mortality	16.6%	10.5%	6.1%	Khan [21]
**Costs**				
Daily Drug Costs	$0.07	$0.12	$0.05	IDPIG [16]
Days on Drug Therapy	168	112	–	MOH [9]
Total Drug Cost for new TB cases	$11.63	$12.90	$1.28	IDPIG [16] b
Cost per Clinic Visit	$0.19	$0.19	–	IDI^c^
Number of Monthly Clinic Visits	6	4	–	MOH [9]

a Treatment success was defined as patients who did not experience failure, relapse, or within-trial mortality. For HIV-negative patients, our definition of treatment success varies slightly from Jindani [7] and Nunn [11] in that we assume that patients with doubtful or doubtfully favorable status are included in the treatment success category.

b Defined as 6HE: $0.0692 times 168 days  =  $11.63; 4HR: $0.1152 times 112 days  =  $12.90.

c Internal analysis conducted at the Infections Diseases Institute in Kampala, Uganda.

## Methods

All analyses were conducted from the perspective of the Ugandan national health care system. In our first model (Model #1, see Figure 1), all patients were considered to be TB treatment naïve and eligible for treatment as new TB cases. In summary, patients received treatment consisting of the fixed-dose regimen (2RHZE) in the intensive phase of TB treatment and were then assigned to 6HE or 4HR at the decision node. We subdivided our model into an HIV-positive and an HIV-negative arm to account for the high prevalence of HIV in TB patients in Uganda (54%) [10]. For HIV-negative patients, the likelihood of a favorable treatment outcome was based on evidence from two randomized clinical trials between the two treatment strategies that were conducted in mostly HIV-negative populations [7], [11]. An unfavorable treatment outcome was defined as the sum of treatment failure, relapse, and within trial mortality. For HIV-positive patients, the likelihood of these three clinical endpoints was based on a systematic review of randomized, controlled trials and cohort studies of rifampicin for either 2 or 6 months in HIV-positive patients with TB. Patients with a favorable treatment outcome only accrued the cost of TB treatment. Patients with unfavorable treatment outcomes (relapse, failure) were assumed to progress to retreatment involving a daily injection of streptomycin for 2 months and concomitant RHZE followed by 1 month of RHZE and 5 months of RHE, as per the WHO and national guidelines [8], [12]. The two retreatment outcomes included in the model were completion of treatment and all-cause mortality, which was varied for HIV-positive and HIV-negative patients requiring re-treatment based on data from a prospective Ugandan cohort [13].

**Table 2:** TB Retreatment and MDR-TB Treatment.

TB Retreatment Efficacy	Basecase	Source Data
% of patients accessing retreatment	100.0%	Assumption^a^
*HIV-negative patients*		
Treatment Success	93.2%	Jones-Lopez [13] b
Treatment Mortality	6.8%	Jones-Lopez [13]
*HIV-positive patients*		
Treatment Success	80.0%	Jones-Lopez [13] b
Treatment Mortality	20.0%	Jones-Lopez [13]
**TB Retreatment Costs**		
Sputum Culture	$48.00	Makerere University^c^
Streptomycin (56 vials)	$5.64	NTLP^d^
Streptomycin administration (56 injections)	$23.80	IDI^e^
6 Clinic Visits	$1.14	MOH [9]
RHZE^f^, if patient <50 kg, 40% of sample (84 days)	$14.04	NTLP^d^
RHZE, if patient >50 kg, 60% of sample (84 days)	$18.72	NTLP^d^
RHE^g^	$15.27	NTLP^d^
Sum	$110.70	
Treatment Costs of Non-surviving Patients	50.0%	Jones-Lopez [13] h
**MDR-TB Treatment**		
**MDR-TB Treatment Efficacy**		
% of patients accessing retreatment	100.0%	Assumption^a^
MDR-TB Prevalence	11.7%	Lukoye [14]
*HIV-negative patients*		
Treatment Success	80.0%	Seung [15] b
Treatment Mortality	20.0%	Seung [15]
*HIV-positive patients*		
Treatment Success	67.9%	Seung [15] b
Treatment Mortality	32.1%	Seung [15]
**MDR-TB Treatment Costs**		
MDR-TB Treatment Costs	$3,355.00	Tupasi [25]
MDR-TB Treatment Costs of Non-surviving Patients	26.2%	Seung [15] i

a The actual proportion of patients that are able to access either re-treatment or MDR-TB treatment is not reported for Uganda. However, we based our model on optimal access to care and varied this parameter in sensitivity analyses.

b The measures of treatment success in our analysis is different from the original publications by Jones-Lopez [13] and Seung [15]. We simplistically assumed that all surviving patients experienced a successful treatment outcome in our model and applied the full cost of TB re-treatment or MDR-TB treatment to surviving patients.

c Department of Medical Microbiology, Makerere University Kampala, Uganda.

d Ugandan National Tuberculosis and Leprosy Program (NTLP) reported in 2010.

e Internal analysis conducted at the Infections Diseases Institute in Kampala, Uganda.

f RHZE, rifampicin, isoniazid, pyrazinamide, ethambutol.

g RHE, rifampicin, isoniazid, ethambutol.

h For HIV-positive and HIV-negative patients combined, the mortality rate in Jones-Lopez [13] was reported as 7, 17, and 14 in the periods of 0–2 months, 2–5 months, and 5–8 months, respectively. Since more detailed information on the timing of deaths was not available, we assumed that these patients would have incurred half of the relevant treatment costs.

i In Seung [15], death occurred after a median 66 days in treatment. Among surviving patients, the median duration of treatment was reported at 252 days, thus, we assumed that non-surviving patients would consume 26.2% (66/252 = 26.2%) of resources relative to surviving patients.

In Model #2, we allowed for the additional option of MDR-TB treatment. Following unfavorable TB treatment of a new case, patients could either progress to retreatment or be treated for multi-drug resistant TB (MDR-TB), based on reported estimates of the local MDR-TB prevalence for previously treated TB patients in Uganda [14]. The MDR-TB treatment outcomes included in the model were either treatment completion or all-cause mortality. The rate of mortality among HIV-negative as well as HIV-positive MDR-TB patients was based on data reported by the Lesotho national MDR-TB program [15].

Based on our models, we estimated the average cost per treated patient, the expected mortality rate by treatment, as well as the cost per life saved. Sensitivity analyses were done by varying select inputs, applying the cost of DOT, varying the rate of MDR-TB in the 4HR arm and the percentage of patients that are able to access MDR-TB treatment. We considered a treatment strategy to be dominant if it was associated with both lower costs as well as lower mortality. For strategies that were associated with higher costs and lower mortality we calculated the cost per life saved. All calculations were performed in Excel 2007 (Microsoft Corporation, Redmond, WA, USA).

**Figure 2:**
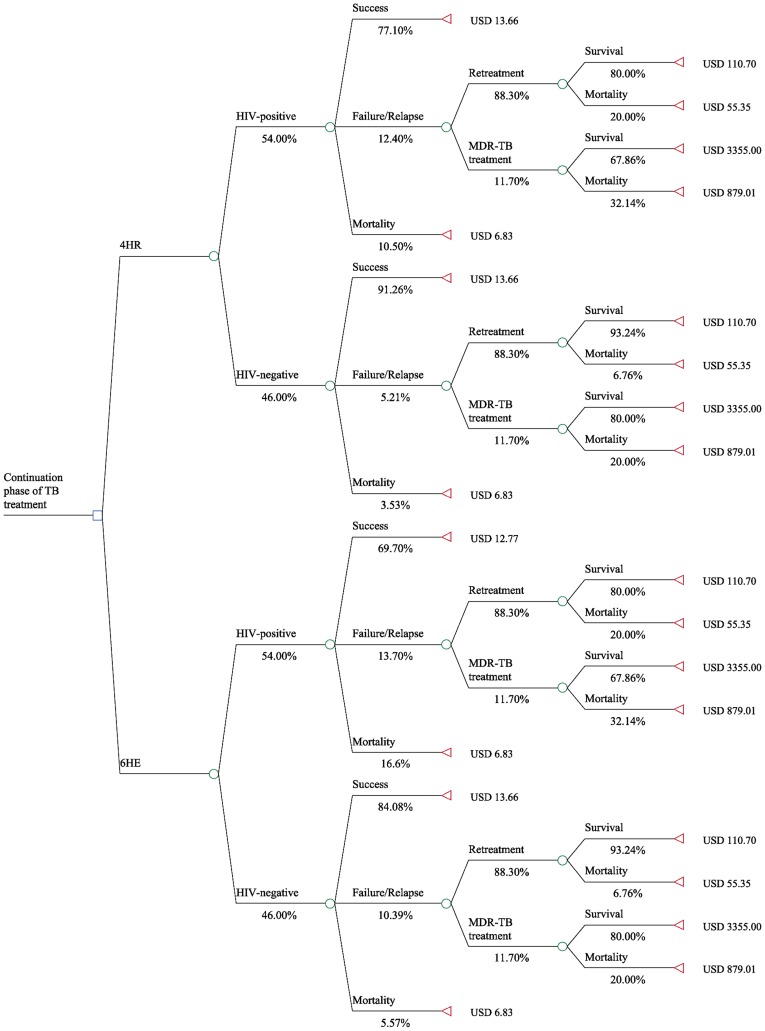
Model #2.

### Costs of Treatment of New TB Cases

Drug costs for treatment of new TB cases in our model were based on published international drug prices (2008), are expressed in United States dollars ($), and are displayed in Table 1 [16]. The price of fixed dose combination HE (150 mg/400 mg) ranged from $0.025–$0.0378 per pill (full range used in sensitivity analyses) and the median listed price of $0.0346 was used throughout the analysis. The total daily drug cost was therefore estimated to be $0.0692, based on daily dosing (2 pills/day). In comparison, the price of fixed dose combination HR (150 mg/300 mg) ranged from $0.0390–$0.0814 per pill, with a median price of $0.0576 and daily drug cost of $0.1152, which were also based on daily dosing (2 pills/day).

In the continuation phase of the treatment of new TB cases, the national TB program calls for 6 monthly clinic visits to monitor for treatment progress and adverse events and for drug refills. The currently used 6HE regimen involves an additional 2 clinic visits compared to the 4HR regimen. An internal analysis was conducted over the course of one week in June 2011 at the integrated TB/HIV clinic at the Infectious Diseases Institute (IDI) in Kampala, Uganda [17], which showed that the average follow-up visit conducted by a nurse in the continuation phase of treatment lasted for a mean of 5 minutes (unpublished data). In the Ugandan health care setting, these patients are routinely seen by a TB nurse at a salary of approximately 1 million Ugandan Shillings (UGX) per month (costs obtained internally from the IDI), which was equivalent to $400 based on the exchange rate when this analysis was conducted in 2011 (1$ = 2,500 UGX). This translated into a cost for a 5-minute visit of 484 UGX, or $0.19.

**Table 3:** Results from Sensitivity Analyses.

				Cost/Life Saved 4HR vs. 6HE			
Sensitivity Analyses	Basecase	Sensitivity Range		Model #1	Model #1	Model #2	Model #2
Treatment Specific Efficacy	Difference	Low	High	Low	High	Low	High
*HIV-negative patients*							
Treatment Failure/Relapse^a^	5.2%	*0.9%*	*16.0%*	$4	Dominant	Dominant	Dominant
Treatment Mortality^a^	2.0%	*0.6%*	*10.8%*	Dominant	Dominant	Dominant	Dominant
*HIV-positive patients*							
Treatment Failure/Relapse^b^	1.3%	−0.1%	6.0%	Dominant	Dominant	Dominant	Dominant
Treatment Mortality^b^	6.1%	3.4%	8.6%	Dominant	Dominant	Dominant	Dominant
**Treatment Specific Costs**							
Total Drug Cost for new TB cases c	$1.28	−$0.74	$5.89	Dominant	$45	Dominant	Dominant
**Other Efficacy Parameters**	**Basecase**	**Low**	**High**	**Low**	**High**	**Low**	**High**
*HIV-negative patients*							
Retreatment Mortality^d^	6.8%	3.4%	10.1%	Dominant	Dominant	Dominant	Dominant
MDR-TB Treatment Mortality	20.0%	10.0%	30.0%	–	–	Dominant	Dominant
*HIV-positive patients*							
Retreatment Mortality^d^	20.0%	10.0%	30.0%	Dominant	Dominant	Dominant	Dominant
MDR-TB Treatment Mortality	32.1%	16.1%	48.2%	–	–	Dominant	Dominant
*% of Patients able to access*							
Retreatment	100.0%	0.0%	50.0%	$20	Dominant	Dominant	Dominant
MDR-TB Treatment	100.0%	0.0%	50.0%	–	–	Dominant	Dominant
*MDR-TB Prevalence*							
Among Previously Treated Patients	11.7%	4.8%	22.6%	–	–	Dominant	Dominant
**Other Cost Parameters**	**Basecase**	**Low**	**High**	**Low**	**High**	**Low**	**High**
Cost per Clinic Visit^e^	$0.19	$0.15	$0.25	Dominant	Dominant	Dominant	Dominant
Retreatment Cost^e^	$110.70	$83.02	$138.37	Dominant	Dominant	Dominant	Dominant
MDR-TB Treatment Cost^e^	3,355.00	2,516.25	4,194.75	–	–	Dominant	Dominant
DOT, Monthly Cost^f^	$0.00	$7.83	$16.32	Dominant	Dominant	Dominant	Dominant
*Treatment Cost of Non-surviving Patients*							
Treatment of new TB cases^e^	50.0%	25.0%	75.0%	Dominant	Dominant	Dominant	Dominant
RetreatmentI^e^	50.0%	25.0%	75.0%	Dominant	Dominant	Dominant	Dominant
MDR-TB	26.2%	15.0%	40.0%	–	–	Dominant	Dominant

a Based on average upper and lower 95% CI of the center adjusted odds ratio of unfavorable status of 6HE vs. 4HR from Jindani [7] and Nunn [11].

b Based on 95% confidence interval from Khan [21].

c Based on the highest and lowest quoted price from IDPIG [16].

d Assuming a 50% variation around the mortality estimate.

e Assuming a 25% variation around this cost estimate.

f Based on confidence interval reported in Aspler [19].

**Figure 3:**
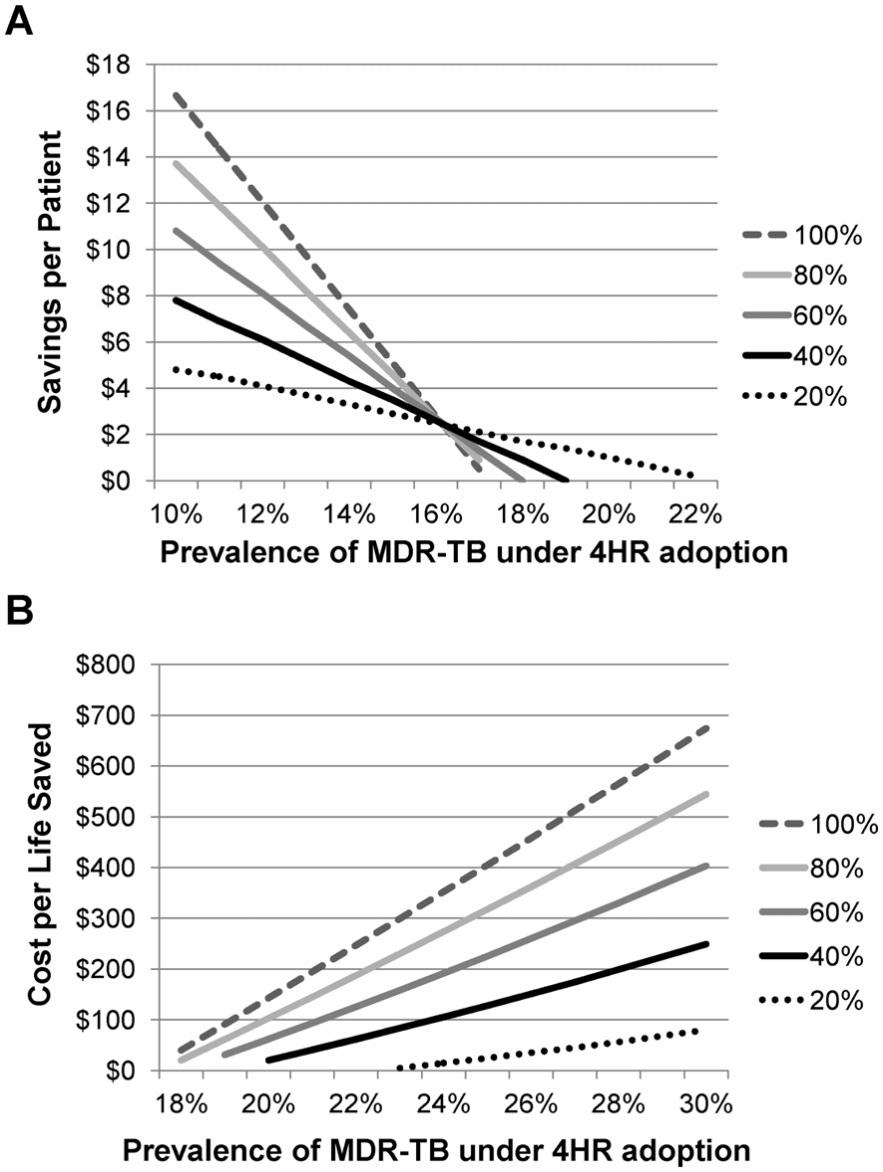
Sensitivity Analysis. (a) Per patient savings by MDR-TB prevalence and 20%–100% access to MDR-TB treatment. (b) Cost/life saved by MDR-TB prevalence and 20%–100% access to MDR-TB treatment.

In Uganda, due to the potential for unfavorable drug-drug interactions, HIV-TB co-infected patients receiving nevirapine (NVP) as part of their combination antiretroviral therapy (cART) regimen are switched to the more expensive efavirenz (EFV) for the duration of rifampicin treatment [18]. However, information obtained from a HIV treatment information center in Uganda (personal communication, S. Zawedde AIDS Treatment Information Centre, the IDI, Makerere University, Kampala, Uganda) and data from our clinic (unpublished) suggest that these patients are rarely switched back to NVP after completing rifampicin treatment. Since patients receiving 6HE remain on EFV-based regimens, we assumed no incremental cART related costs in our 4HR cohort. Lastly, even though directly observed treatment (DOT) is currently not the standard of care in Uganda, we also explored the monthly cost of clinic-based DOT in sensitivity analyses, based on cost-data reported for Zambia [19].

### Retreatment Costs

Retreatment drug costs were based on actual procurement costs incurred by the National Tuberculosis and Leprosy Program (NTLP) in Uganda in 2010 (Table 2). The average procurement cost per vial of streptomycin was reported at $0.10. We also included the cost of streptomycin administration, assuming a daily 5-minute clinic visit at a cost of $0.19. The cost of a syringe ($0.09), antiseptic iodine solution ($0.02), gauze ($0.09), and sticking plaster ($0.03), were based on institutional costs from a previous analysis [20], yielding a total cost per injection of $0.53 As per the national guidelines, samples (sputum, lymph node aspirates) of patients initiating retreatment are to be sent for culture and drug and sensitivity testing [12]. As national data on which proportion of retreatment patients are able to produce samples was not available, we also assumed that sputum cultures were obtained from all retreatment patients at a cost of $48 per culture (Department of Medical Microbiology, Makerere University Kampala, Uganda).

The NTLP lists the price of fixed dose combination RHZE (150 mg/75 mg/400 mg/275 mg) used in the first 3 months of retreatment at $0.0557 per pill [16]. However, dosing is weight-dependent and involves 3 tablets a day in patients weighing less than 50 kg and 4 tablets a day in patients weighing more than 50 kg [12], from which we generated a daily cost of $0.17 and $0.22, respectively. An internal analysis of patients initiated on TB therapy at the IDI in 2009 suggested that the proportion of baseline weights below and above 50 kg was 40% and 60%, respectively, which is the ratio we used to generate the weight-adjusted cost of RHZE in our model. In the remaining 5 months of retreatment treatment, fixed dose combination RHE (150 mg/75 mg/275 mg) was listed at $0.0364 per pill and involved 3 tablets a day, resulting in a daily drug cost of $0.1091 (NTLP). For the proportion of patients with a mortality outcome in our model, 50% of the retreatment drug costs were applied.

### Treatment Efficacy

Treatment efficacy was stratified by HIV-status. In mostly HIV-negative patients, the clinical efficacy of 6HE versus 4HR in the continuation phase of treatment for new TB cases has been evaluated in two prospective, randomized clinical trials [7], [11]. In these two studies combined, a total of 627 patients in the two 6HE arms and 629 patients in the two 4HR arms were clinically evaluable at the end of follow-up. The total number of deaths during the assessment period was 37 (6HE) and 23 (4HR). Using the sum of clinically evaluable patients and deaths in the denominator, treatment failure and relapse were 69 (10.4%) in the two 6HE arms and 34 (5.2%) in the two 4HR arms, respectively. This difference was statistically significant in both trials individually (*P = *0.006 and *P = *0.002, respectively). In sensitivity analyses, we used the average of the two 95% confidence intervals around these estimates.

Efficacy estimates in HIV-positive patients, were based on a systematic review of treatment outcomes among HIV-infected patients with TB [21]. We used the pooled event rate of short-term (2 months) rifampicin treatment as a proxy to inform modeled outcomes in the 6HE arm of our model and the longer-term (6 months) rifampicin treatment as a proxy for our 4HR arm. The sum of the pooled failure and relapse event rates were used to define unfavorable treatment outcomes, which added up to 13.7% in the 6HE arm and 12.4% in the 4HR arm of our model; the pooled mortality rate was reported at 16.6% and 10.5%, respectively. The 95% confidence intervals around the pooled event rates served as inputs in sensitivity analyses. The final efficacy parameter we included was the mortality rate of patients on the retreatment regimen, which ranged from 6.8% in HIV-negative patients to 20.0% in HIV-positive patients [13] and this was varied by −/+50% in sensitivity analyses.

### Multi-Drug Resistant TB (MDR-TB)

In model #2, we evaluated the impact of MDR-TB. The prevalence of MDR-TB in Uganda is currently estimated at in 11.7% in patients previously treated for TB [22]. MDR-TB treatment involves a combination of 4 active drugs from the following: first-line drugs to which the TB strain remains susceptible, injectable aminoglycosides, fluoroquinolones, bacteriostatic second-line drugs and other more toxic alternatives [23]. Given the toxicity of the MDR-TB treatment, patients must be followed closely and have a higher risk of treatment failure [24]. MDR-TB treatment has been associated with average treatment costs among surviving and non-surviving patients as high as $3,355 [25].

We assumed that the probability of a favorable outcome remained unchanged in the 4HR as well as the 6HE treatment arm compared to model #1. We added a chance node following an unfavorable treatment outcome for a new TB case, where 11.7% required treatment for MDR-TB (Figure 2). We assumed that no patients were found to have MDR-TB before starting treatment for new TB cases and that all patients requiring treatment for MDR-TB were able to access it. The remaining 88.3% (100%−11.7%) were assumed to initiate standard retreatment treatment. Among treated patients, MDR-TB related mortality was reported at 32.1% in patients co-infected with HIV and 20.0% in HIV-negative patients [15]. We varied this mortality rate by −/+50% in the sensitivity analyses.

To account for the possibility that the rate of MDR-TB may increase if 4HR were to replace 6HE, we ran additional sensitivity analyses where we varied the rate of MDR-TB among previously treated patients in the 4HR treatment arm only. We assumed that the rate of MDR-TB among patients in the 6HE treatment arm would remain unchanged at 11.7% [13]. In this calculation, we also varied the percentage of patients that were assumed to be able to access MDR-TB treatment in Uganda from 20%–100%; patients who were assumed not to be able to access MDR-TB treatment did not incur any cost and had a mortality outcome in the model. For increasing rates of MDR-TB, we evaluated the average savings per patient associated with 4HR as well as the cost per life saved in cases where 4HR was the more expensive option.

## Results

### Model #1

Model #1 structure and results are displayed in Figure 1. The average total cost of treatment of a new TB case, including drug costs and clinic visits, was $12.77 for 6HE and $13.66 for 4HR. The average total cost of a completed course of retreatment regimen, including the cost of drugs, streptomycin administration, clinic visits and TB sputum culture, and adjusted for baseline weight was $110.70. Hence, the expected average cost of TB treatment in the continuation phase was $26.07 for 6HE, which compared to $23.64 for 4HR. The expected cost savings associated with 4HR were $2.42 per patient. Furthermore, our model predicted an expected mortality rate of 13.3% associated with 6HE treatment and 8.8% associated with 4HR treatment. Therefore, we did not calculate an incremental cost-effectiveness ratio, since 4HR was associated with both lower cost as well as lower expected mortality in the base case (e.g. dominant strategy).

### Model #2

Model #2 structure and results are displayed in Figure 2. Including treatment for MDR-TB as an additional outcome for previously treated patients with unfavorable outcomes increased the average cost to $65.86 for 6HE and to $53.12 for 4HR. The cost difference between the 6HE and 4HR treatment options increased to $12.74 per patient. The expected mortality increased slightly to 13.5% and 8.9%, respectively, and 4HR continued to be the dominant treatment strategy (e.g. lower cost and lower mortality) relative to 6HE.

### Sensitivity Analyses

Results from our one-way sensitivity analyses are displayed in Table 3. We found that 4HR generally dominated 6HE over a wide range of inputs in both models. In the three cases where 4HR did not dominate 6HE, it was associated with cost-effectiveness thresholds ranging from $4–$45 per life saved. We also found that a higher rate of MDR-TB in the 4HR arm of our model diminished the average cost-savings per patient. Depending on the proportion of patients that were assumed to be able to access MDR-TB treatment, 4HR was associated with zero cost-savings relative to 6HE if the MDR-TB prevalence increased from 11.7% to 17%–22% (see Figure 3a). At higher rates, 4HR was expected to increase the average treatment cost, but was associated with a cost per life saved of $80–$674 even at resistance rates as high as 30% (see Figure 3b).

## Discussion

In our 2 models comparing 4HR to 6HE, 4HR is associated with a lower expected mortality rate as well as lower total cost. The first model does not consider the cost of MDR-TB cases, but rather just the cost of the TB treatment regimens in new patients and in those requiring retreatment. In estimates from the WHO, a decreased cost of $4–10 was estimated [8]. Our estimate of $2.42 per patient falls slightly below this range. More importantly, because the rate of relapse or failure is higher in patients who take 6HE based on data from clinical trials, this increased likelihood of an unfavorable outcome may lead to an increased risk of MDR-TB. When we considered the cost of MDR-TB in our second model, the cost difference was even larger ($12.74 per patient). Thus, using the WHO estimate of 40,000 new TB cases annually in Uganda, our model results suggest that adopting 4HR may reduce the annual TB-related mortality burden by ∼1,800 patients and may lead to expected savings of approximately $100,000–$500,000 per year to the Ugandan national health care system.

Our estimate of the number of cases generated is conservative because we do not model the cost of the patients who also have isoniazid mono-resistance and who are often cured with 4HR in the continuation phase, but have an increased rate of treatment failure with 6HE [26]. Furthermore, we do not consider additional 4HR-related benefits; TB treatment completion rates in the regimen that is 2 months shorter will likely be higher. Also, if the Ugandan TB treatment guidelines for DOT are fully implemented, the results from our sensitivity analysis suggest that the shorter duration of therapy with 4HR would be expected to result in even greater cost-savings relative to 6HE.

Our results are largely driven by the lower mortality rate as well as the lower failure or relapse rate associated with 4HR. In our model, MDR-TB can only occur in previously treated patients in our model. In sensitivity analyses, we found that even if the MDR-TB rate among previously treated patients were to increase with 4HR (although data from one systematic review suggests higher TB drug resistance rates with 6HE [26]), total treatment costs would still be lower with 4HR if the MDR-TB rate were to double because significantly fewer patients would require re-treatment due to the lower relapse rates with this shorter regimen. Furthermore, if the rate of MDR-TB were to increase as high as 30%, 4HR would still be associated with a lower expected mortality rate at an acceptable cost per life saved of $80–$674 depending on the proportion of patients with access to treatment; the mortality rate associated with 4HR for new TB cases is reduced by 2% in HIV-negative patients and 6.1% in HIV-positive patients. Since this reduction applies to all patients that enter the model, it has a disproportionately larger impact on expected mortality than the rate of MDR-TB, which only applies to the subset of patients that require re-treatment.

One of the limitations of our study is that our cost estimates are from the perspective of the Ugandan national health care system with the cost of clinic procedures and cultures based on local rates and a rate of HIV co-infection of 54%. Similar analyses in other countries still using the 6HE regimen should be conducted to understand the generalizability of our results. In addition, our analysis was based on clinical efficacy results as obtained in several randomized controlled clinical trials and cohort studies and actual effectiveness in a real world setting with arguably lower compliance rates could be different. Other limitations include: 1) that the majority of the aforementioned studies were not conducted in Uganda and that these studies did not evaluate whether the numerical differences in mortality between 4HR and 6HE were statistically significant, 2) we did not evaluate the impact of increasing rates of MDR-TB in newly treated patients. Lastly, Green Light Committee (GLC) MDR treatment regimens may allow for significantly lower costs associated with MDR treatment. Although MDR-TB treatment is approved by the GLC for Uganda, drugs are still not available and no patients are currently on treatment through the national program. Finally, despite increased emphasis on cost effectiveness to inform health policy decision-making and coverage decisions, cost effectiveness should supplement, not replace clinical effectiveness evaluations.

In summary, a transition to the strongly recommended continuation phase 4HR regimen is associated with lower costs, lower mortality, a lower overall risk for relapse and, therefore, a reduced need for retreatment. The Ugandan national program will need to continue to enhance health systems to allow for better monitoring of drug compliance to implement rifampicin throughout the course of TB treatment, but the benefit of fewer cases of retreatment and drug resistant TB as well as cost savings, provide compelling arguments for change.
